# Supplementary material for: Isolated Effects of Plasma Freezing versus Thawing on Metabolite Stability
Source: Metabolites. 2022 Nov 11;12(11):1098. doi: 10.3390/metabo12111098 (PMC9693613; doi:10.3390/metabo12111098)
Supplement: Supplementary file 1 [file metabolites-12-01098-s001.zip › FigureS1_221031_SupplementalPCA_FreezeThawPaper.pdf]

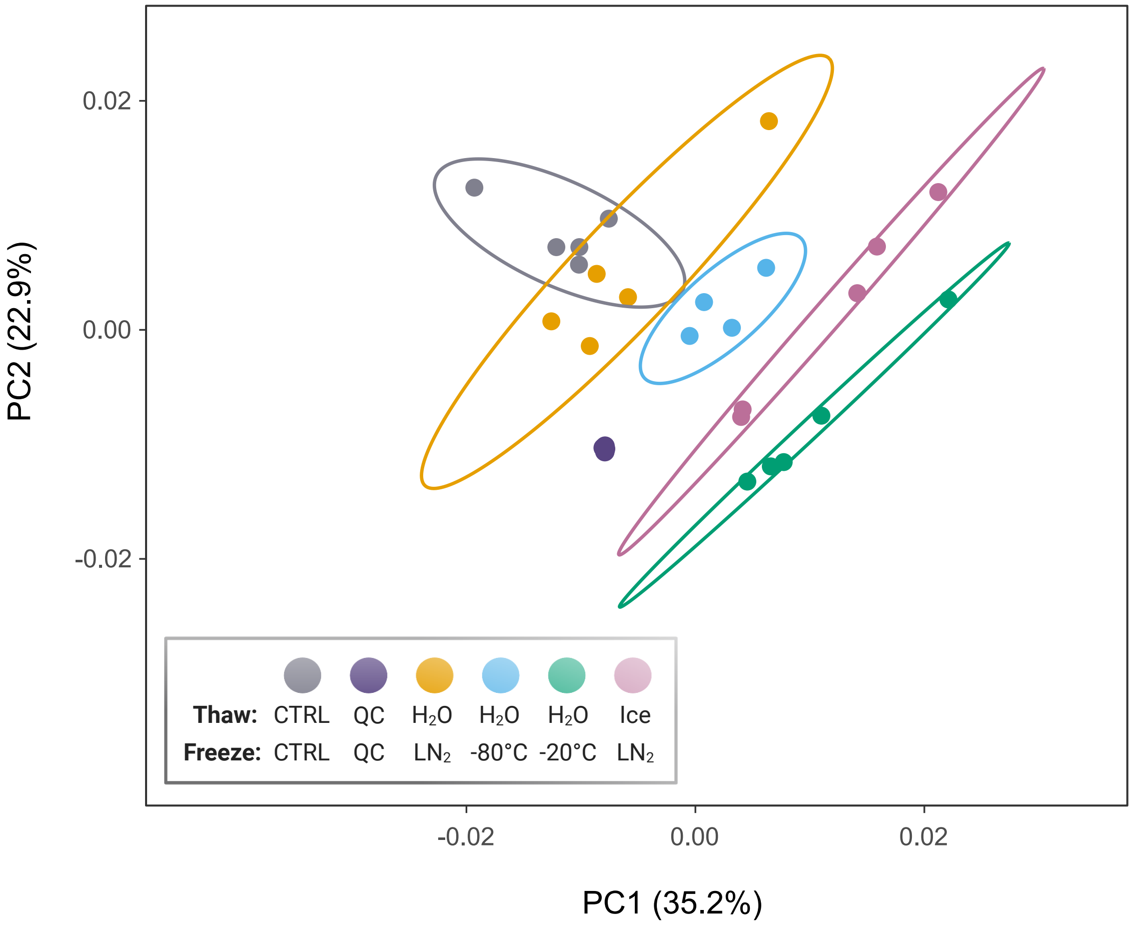

Figure S1. PCA of NOREVA-corrected, ratiometrically normalized peak signal intensities for each treatment group and the quality control (QC) samples.
